# Yoga & Cancer Interventions: A Review of the Clinical Significance of Patient Reported Outcomes for Cancer Survivors

**DOI:** 10.1155/2012/642576

**Published:** 2012-10-17

**Authors:** S. Nicole Culos-Reed, Michael J. Mackenzie, Stephanie J. Sohl, Michelle T. Jesse, Ashley N. Ross Zahavich, Suzanne C. Danhauer

**Affiliations:** ^1^Faculty of Kinesiology, University of Calgary, 2500 University Drive NW, Calgary, AB, Canada T2N 1N4; ^2^Department of Psychosocial Resources, Tom Baker Cancer Centre, Canada; ^3^Department of Oncology, Faculty of Medicine, University of Calgary, Canada; ^4^Department of Social Sciences and Health Policy, Division of Public Health Sciences, Wake Forest School of Medicine, Medical Center Boulevard, Winston-Salem, NC 27157-1063, USA; ^5^Department of Internal Medicine, Section on Hematology and Oncology, Wake Forest School of Medicine, Medical Center Boulevard, Winston-Salem, NC 27157-1063, USA; ^6^Department of Psychology, University of North Carolina at Charlotte, Charlotte, NC 28223-0001, USA

## Abstract

Limited research suggests yoga may be a viable gentle physical activity option with a variety of health-related quality of life, psychosocial and symptom management benefits. The purpose of this review was to determine the clinical significance of patient-reported outcomes from yoga interventions conducted with cancer survivors. A total of 25 published yoga intervention studies for cancer survivors from 2004–2011 had patient-reported outcomes, including quality of life, psychosocial or symptom measures. Thirteen of these studies met the necessary criteria to assess clinical significance. Clinical significance for each of the outcomes of interest was examined based on 1 standard error of the measurement, 0.5 standard deviation, and relative comparative effect sizes and their respective confidence intervals. This review describes in detail these patient-reported outcomes, how they were obtained, their relative clinical significance and implications for both clinical and research settings. Overall, clinically significant changes in patient-reported outcomes suggest that yoga interventions hold promise for improving cancer survivors' well-being. This research overview provides new directions for examining how clinical significance can provide a unique context for describing changes in patient-reported outcomes from yoga interventions. Researchers are encouraged to employ indices of clinical significance in the interpretation and discussion of results from yoga studies.

## 1. Introduction

Physical activity is widely accepted as beneficial for cancer survivors both on and off treatment [1]. It is recommended that adult survivors engage in the same amount of physical activity as healthy older adults, with adaptations made as necessary [2]. This recommendation is supported by a substantive amount of evidence documenting the beneficial effects of PA on quality of life, psychosocial, and physical/symptom outcomes [1]. Within this growing body of physical activity and cancer research, there have been calls to examine modes of physical activity from the area of complementary medicine as a means of improving participation rates and long-term adherence [3]. This approach is particularly important when considering potential additional barriers experienced during a cancer diagnosis, its subsequent treatments, and ongoing recovery [3, 4].

Yoga is quickly emerging as an important complementary medicine therapeutic approach for cancer survivors. Ross and Thomas' [5] review of exercise and yoga highlights that yoga is as beneficial as more traditional types of physical activity at improving a variety of health-related outcome measures (with the exception of physical fitness outcomes) in both healthy individuals and those with various health conditions (e.g., cancer). It concludes that yoga is a potentially beneficial gentle form of physical activity for cancer survivors and continues to receive growing research attention in cancer populations [5].

Additionally, four recent reviews have summarized findings specifically on yoga for cancer [6–9]. Smith and Pukall [6] conducted the first systematic review of the yoga and cancer research to date. This review reported both the characteristics and effect sizes of ten studies, including six randomized controlled trials. The authors documented large variability across studies (i.e., yoga type, population, sample size, and intervention duration) as well as methodological limitations. Despite these issues, generally positive results, especially in terms of psychological outcomes, were noted based on a calculation of effect sizes and narrative summary of reported results. In addition, a recently published meta-analysis aimed to determine effects of yoga on quality of life, psychological, and physical health in cancer survivors [7]. Ten studies were examined, including both yoga and mindfulness-based stress reduction (MBSR) interventions, due to the inclusion of yoga in MBSR. Although the results are preliminary and should be interpreted cautiously, intervention groups showed significantly greater improvements in psychological health than waitlist or supportive therapy control groups [7]. Results were inconclusive for quality of life and physical health outcomes. Undoubtedly, research examining yoga for cancer survivors is in its infancy. However, as research in this area continues to rapidly grow and yoga is increasingly integrated into cancer care, it is imperative the clinical benefits of yoga for cancer survivors are better understood and emphasized within clinical and posttreatment survivorship care. Thus the focus of the current study is to better determine the implications of the existing research on yoga for cancer survivors by evaluating additional indicators of clinical significance.

Reviews of the research on yoga for cancer survivors to date have relied on the interpretation of effect sizes and subjective observation of trends. Subjective interpretation of trends is often heavily influenced by reporting of the *P*-value, which is commonly used as a statistical indicator of the benefits of an intervention. However, the *P*-value, often set at  .05, indicates only whether or not observed changes are large enough to conclude that such differences were not caused by chance [24]. In addition, the *P*-value is largely contingent on sample size, with larger studies more likely to report statistical significance. Clinical significance, or minimal clinically important difference (MCID), may not involve statistical significance [25] and is considered a marker of the effectiveness of interventions that takes into account the practical importance of treatment effects [26]. Clinical significance also gives meaning to observed changes in terms of their implications for patient care [27, 28]. In the case of our current paper, clinical significance may also be used as a comparative metric of treatment effects between studies.

There are a number of widely accepted assessments of the clinical significance of change in an intervention, using both anchor-based (i.e., clinical) and distribution-based (i.e., statistical) assessments [26, 28]. Anchor-based approaches are methods that relate change to an external event, rating, or condition, while distribution-based methods link clinical significance to a statistical parameter of group or individual data [28]. Examination of the yoga and cancer intervention literature reveals minimal reporting of anchor-based metrics. Given this an anchor-based anchor-based approach for understanding clinical significance was not employed in the current overview of the literature.

Commonly reported distribution-based methods include one standard error of the measurement (1 SEM), 0.5 standard deviation (0.5 SD), and effect sizes. The SEM provides an index that incorporates both the variation and reliability of a sample on any measure expressed in the original metric of the measure it describes [29]. When examining the mean difference from pre- to postintervention, a value greater than 1 SEM is considered clinically significant. One SEM has been shown to consistently correlate with anchor-based measures of important difference and with an effect size of 0.5, if the reported reliability is ≥0.75 [29]. Caution must be used in interpreting the SEM, as this criterion is often predetermined from other studies and may not be derived directly from the study sample.

Second, the assessment of the clinical significance is often determined using 0.5 SD of the baseline measure as a “rule of thumb” or guideline [30, 31]. Specifically, pre-post mean differences larger than 0.5 SD on that scale are considered clinically significant. It has been suggested [26] the 0.5 SD is best used as an indicator of “meaningful difference” versus “minimal difference,” suggesting a change that cannot be ignored. Third, effect size (ES) is a simple way of quantifying the differences between two groups, and by association, how large a clinical response is observed [32]. Helpful exploratory criteria are Cohen's convention of 0.20 for a small effect, 0.50 for a moderate effect, and 0.80 for a large effect [33]. Of note, 0.5 SD of the preintervention score (in the case of pre-post within subjects) or 0.5 of control baseline (in the case of between-groups) can equate with 0.50 ES (provided these measures are calculated using these same baseline measures as the denominator).

Finally confidence intervals (CI) provide a range of possible scores for a population parameter. Thus, in the case of the oft reported 95% CI, we can be 95% confident that our CI includes the population parameter [34]. The width of the CI reflects the precision of the data, with more narrow confidence intervals being more precise. CIs are not just a surrogate for *P* value reported statistical significance, as they also give us a window into both the size of the difference and precision in estimating the true difference [35].

In all cases, it is important not to use these indices (1 SEM, 0.5 SD, ES, CI) in the way *P* values often are: as arbitrary cut-off values of a study's given significance [36]. Rather, these values should be used concurrently to triangulate on and describe the range of one's findings, their relative magnitude of effect, and generalizability. Such a thorough examination of the clinical significance of published studies on yoga for cancer survivors has not been completed to date. The purpose of this paper was to clarify how findings from yoga interventions for cancer survivors should be interpreted by implementing multiple methods for calculating clinical significance of patient-reported outcomes, including measures of general quality of life, psychosocial factors, and symptoms. Interpretation of the studies' findings consisted of examining both within group pre-post change in both control and pre-post design studies, as well as determining the magnitude of difference between treatment and control groups in control design studies. Factors that may influence clinical significance related to the study design, sample, specific intervention, and method of measurement were also documented.

## 2. Methods

### 2.1. Selection of Publications

This literature review of yoga interventions for cancer survivors, both on and off treatment, was completed in July 2011. To this end, a systematic search was conducted of the relevant databases, including PubMed, PsychInfo, Medline, Annotated Bibliography of Indian Medicine, Cochrane Library, Web of Science, and Google Scholar. Key search terms included *yoga, cancer, survivor, patient, intervention, quality of life, well-being, clinical significance, important change(s), important difference(s), and/or patient reported outcomes*. The following inclusion criteria were applied to the search: (1) must include a yoga intervention; (2) have a sample of exclusively adult cancer survivors (on or off treatment); (3) report at least pre- and post-intervention assessments; (4) include patient-reported outcomes related to quality of life, psychosocial, and/or symptom outcomes. Publications were excluded if they incorporated intervention components in addition to yoga (e.g., MBSR, comprehensive lifestyle change programs, physiotherapy), were published in a non-peer reviewed journal, or were not written in English.

### 2.2. Data Synthesis

The common indices of clinical significance used in the current overview were (1) 1 standard error of the measurement (1 SEM); (2) 0.5 standard deviation (0.5 SD); (3) both within and between-group difference effect sizes (ES: Hedge's *g*) and their respective confidence intervals (CI). In order to examine the clinical significance of findings for each yoga intervention study, both pre-post means and the SDs or SEs had to be available in order to calculate 1 SEM, 0.5 SD, ES, and CI. No follow-up time periods (if included) were analyzed, and only published data were accessed. From the initial 19 eligible publications, a further six were dropped because (a) SDs or SEs were not available; or (b) the same data were reported in duplicate publications. Data were extracted to examine changes pre-post intervention in both the yoga and control groups, as well as the relative difference between groups in the case of control design studies. Clinical significance was determined using the following criteria. First, the SEM was calculated as (pre-intervention SD ∗  $\sqrt{1}$ − Cronbach's alpha). If the Cronbach's alpha was not cited in the study it was derived from either a clinically relevant population, or from prior psychometric evaluations of the instrument. Second, 0.5 SD values were calculated from the baseline SD. Third, ES were calculated using Hedges g for both within-groups effects (post-pre/pooled SD) and between-groups effects (treatment mean difference-control mean difference/pooled change SD). These effects were interpreted following Cohen's convention of small (0.2), medium (0.5), and large (0.8) [33]. Fourth, CIs were calculated for both treatment and control within-subjects mean differences, between-groups mean differences, and their accompanying ES. ES and CIs were calculated using Comprehensive Meta-Analysis (Version 2) software [37]. Pre-post differences were evaluated based on their respective 1 SEM, 0.5 SD ES, and CI, while between-group differences were evaluated based on the control group 1 SEM, 0.5 SD, and the between-groups calculated ES and CI. Finally, given the aforementioned correlation between 1 SEM and an effect size of 0.5 if the reported reliability is ≥0.75 [29], the Pearson product-moment correlation coefficients were calculated to determine the strength of the linear dependence between the 1 SEM and 0.5 SD. As our primary purpose was to evaluate patient-reported outcomes in the yoga literature and given the comparatively small number of studies, no computation of study quality was completed in the current paper. In addition, given our focus on a number of different indices of clinical significance, the small number of studies and heterogeneity in terms of type of yoga, dose response, length of intervention, and outcome measures, average effect sizes across studies were not calculated nor meta-analyses performed. Although we intended that the literature search be inclusive of all existing studies, it did not follow other methods necessary to be considered a systematic review so as not to be redundant with other recent publications [6, 7].

## 3. Results

### 3.1. Description of Studies

The initial literature search resulted in a total of 25 publications. A review of abstracts reduced the final number to 19 publications based on the inclusion and exclusion criteria. Of the 19 publications reviewed, 14 contained enough data to be included in this paper and are summarized in Table 1. Note that Vadiraja et al. [16, 17] are from the same study sample and are therefore reported as one study. The resulting 13 studies included a variety of yoga interventions (with respect to type of yoga and duration), cancer types, and timing and content of assessments. Seven of the 13 studies employed a randomized controlled trial design, including a control group for comparison to the yoga intervention (*n* = 6 with a waitlist control group, *n* = 1 with an active control group). Six studies were single-group pre-post designs.  Table 2 details all study results.

#### 3.1.1. Study Designs

In the randomized controlled trial studies, mean age ranged from 46–60 years. Sample size in the treatment group at time 2 ranged from 13–45 participants. Cancer diagnoses were comprised primarily of breast cancer, with one study focused on lymphoma. Many participants were on active treatment or, in some cases, 3 months or more posttreatment. In the case of Moadel et al. [15] we reported on their secondary analysis that focused on women with breast cancer who were not receiving chemotherapy. Yoga classes were 60–90 minutes in length, and programs lasted 6 to 26 weeks. Yoga styles included Hatha, Integral, Iyengar, Tibetan, Viniyoga, and Vivekananda. The majority of control designs employed a waitlist control group except the Vadiraja study [16, 17], which utilized an active supportive therapy with education control group.

In the single group design studies, mean age ranged from 41–59 years. Sample size at time 2 ranged from 10 to 43 participants. Cancer diagnoses were comprised primarily of breast cancer, with one study focused on ovarian cancer. Many participants were on active treatment or, in some cases, six months or more posttreatment. Yoga classes were 60–90 minutes in length and programs lasted 6 to 10 weeks. Yoga styles included Integral, Iyengar, and “classical” yoga.

#### 3.1.2. Instruments Reviewed

A total of 18 different instruments, assessing patient-reported outcomes in health-related quality of life (HRQL; six instruments), psychosocial (eight instruments), and symptom domains (four instruments) were reviewed. We acknowledge that there is some overlap in HRQL and psychosocial constructs. For the sake of simplicity and parsimony, we left HRQL subscales in the HRQL category even if they capture psychosocial constructs such as social or emotional well-being. Further, in order to minimize the number of categories of patient-reported outcomes, we included spiritual well-being in the psychosocial category.  Table 2 presents a summary of the clinical significance for the patient-reported outcomes in the 13 reviewed studies and is divided into control design and pre-post single group design studies. Each table further distinguishes between patient, reported outcome categories: HRQL, Psychosocial, and Symptom variables.

### 3.2. Quality of Life Outcomes

#### 3.2.1. Overall HRQL

Two control design studies, Danhauer et al. [13] and Culos-Reed et al. [12], met the 1 SEM and 0.5 SD criteria both pre-post and between yoga intervention and waitlist control. Medium between-group ES, ranged from 0.49 (95% CI −0.25, 1.24; *P* = NS) [13] to 0.67 (95% CI 0.01, 1.32; *P* < .05) [12]. Moadel et al. [15], while showing no evidence of clinically significant change pre-post, met the 1 SEM criteria with an overall small ES of 0.43 (95% CI −0.05, 0.91; *P* = NS), reflecting improvement in overall HRQL in the yoga group versus a worsening in the control group. Littman et al. [14] showed no significant differences either pre-post or between-groups. In single group design studies [18–23] clinically significant outcomes ranged from a small ES of 0.39 (95% CI −0.08, 0.86; *P* = NS) meeting the 1 SEM criteria [22], to a large ES of 0.81 (95% CI 0.14, 1.48; *P* < .05) [21], that met both 1 SEM and 0.5 SD criteria. Interestingly, overall HRQL in Danhauer et al. [19], while statistically significant (*P* < .05) with a small ES of 0.32 (95% CI 0.02, 0.63), met neither the 1 SEM nor 0.5 SD criteria.

#### 3.2.2. Physical HRQL

Only two control design studies, Chandwani et al. and Danhauer et al. [10, 13], showed evidence of clinically significant differences. While both studies did not exhibit clinically significant pre-post changes, both studies showed clinically significant effects between groups that met the 1 SEM criteria, with small ES, ranging from 0.36 ES (95% CI −0.38, 1.10; *P* = NS) [13] to 0.46 ES (95% CI −0.06, 0.97; *P* = NS) )[10]. Interestingly, although there was clinically significant change on the FACT physical wellbeing subscale in Danhauer et al. [13], there was no significant change on the SF-12 physical wellbeing subscale. Several other control design studies [14–16] exhibited no clinically significant differences in physical HRQL, either pre-post or between groups. Within the single-group design studies, only the Ülger and Yağlı study [23] showed a large ES of −0.92 (95% CI −1.43, −0.41; *P* < .001) and met both the 1 SEM and 0.5 SD criteria, indicating significant improvements in physical HRQL pre-post yoga intervention. Speed-Andrews et al. [22] met the 1 SEM criteria with a small ES of 0.32 (95% CI −0.14, 0.79; *P* = NS). Interestingly, Danhauer et al. [19] met neither 1 SEM nor 0.5 SD but was statistically significant (*P* < .05) with an effect size of 0.38 (95% CI 0.07, 0.68) on the FACT physical wellbeing subscale. There was no significant change on the SF-12 physical wellbeing subscale.

#### 3.2.3. Mental HRQL

Large effects were seen in control group designs for Danhauer et al. [13] with pre-post and between-group mean differences meeting both 1 SEM and 0.5 SD criteria, with a between group ES of 1.00 (95% CI 0.22, 1.78; *P* < .01). Vadiraja et al. [16] did not meet the 1 SEM or 0.5 SD criteria but reported a statistically significant (*P* < .05) ES of 0.30 (95% CI 0.00, 0.61) pre-post yoga intervention. There were no clinically significant differences between groups. There were also no significant differences either pre-post or between groups in Chandwani et al. [10]. In single group designs, Danhauer et al. [19] pre-post differences did not meet the 1 SEM or 0.5 SD criteria but reported a statistically significant (*P* < .01) ES of 0.41 (95% CI 0.11, 0.72). Speed Andrews et al. [22] demonstrated a small clinically significant difference meeting the 1 SEM criteria with an effect size of 0.45 (95% CI −0.03, 0.93; *P* = NS).

#### 3.2.4. Emotional HRQL

Values ranged from meeting both the 1 SEM and 0.5 SD criteria pre-post and between groups, with ES from 0.53 (95% CI 0.07, 0.99; *P* < .05) [16], to 0.61(95% CI −0.15, 1.35; *P* = NS) [13], between groups. There were no clinically significant differences pre-post for either Moadel et al. [15] or Culos-Reed et al. [12], however both exhibited between-group differences. Culos-Reed et al.[12]exhibited a small ES of 0.48 (95% CI −0.16, 1.13; *P* = NS) between groups that met both the 1 SEM and 0.5 SD criteria. Moadel et al. [15] met neither 1 SEM nor 0.5 SD criteria but demonstrated a small statistically significant (*P* < .01) pre-post ES of 0.40 (95% CI 0.10, 0.70). There were no differences between groups. Littman et al. [14] demonstrated no clinically significant differences either pre-post or between groups. Within single subject designs, Ülger and Yağlı [23] reported large pre-post differences that met both 1 SEM and 0.5 SD with an effect size of −1.15 (95% CI −1.70, −0.60; *P* < .001). There was no clinically significant differences pre-post yoga intervention for either Danhauer et al. [19] or Speed-Andrews et al. [22].

#### 3.2.5. Social HRQL

The majority of control design studies [13, 14, 16] report no clinically significant differences either pre-post or between yoga and waitlist control groups. Interestingly, Moadel et al. [15] demonstrated a moderate, clinically significant decline in the waitlist control group, with an ES of −0.60 (95% CI −1.00, −0.19; *P* < .01). This led to a moderate between group difference of 0.60 ES (95% CI 0.11, 1.09; *P* < .05), which met both the 1 SEM and 0.5 SD criteria, indicating a relative improvement in social HRQL for the yoga group versus a worsening of the waitlist control group. Within the single group designs, Ülger and Yağlı [23] demonstrated a moderate clinically significant difference of −0.74 ES (95% CI −1.22, −0.26; *P* < .01), meeting both the 1 SEM and 0.5 SD criteria, while Danhauer et al. [19] and Speed-Andrews et al. [22] demonstrated no clinically significant differences in social HRQL pre-post yoga intervention.

#### 3.2.6. Functional HRQL

Only Danhauer et al. [13] reported pre-post improvement in Functional HRQL, with a moderate between-group ES of 0.58 (95% CI −0.17, 1.34; *P* = NS) that met both 1 SEM and 0.5 SD criteria. Littman, Moadel, and Vadiraja [14–16], indicated no clinically significant differences either pre-post or between yoga and waitlist control groups. Similarly no clinically significant differences are reported pre-post within single group designs [19, 22].

### 3.3. Psychosocial Outcomes

#### 3.3.1. Depression

Clinically significant differences ranged from small, with a between-groups ES of −0.39 (95% CI −1.04, 0.25; *P* = NS), that met the 1 SEM criteria [12] to medium, with a between groups ES of −0.69 (95% CI −1.45, 0.06; *P* = NS), meeting both 1 SEM and 0.5 SD criteria [13]. Interestingly in the case of Vadiraja et al. [17] large clinically significant differences within the yoga group, −0.89 ES (95% CI −1.25, −0.54; *P* < .001), and small clinically significant differences within the supportive therapy control group, −0.40 ES (95% CI −0.74, −0.05; *P* < .05), indicated significant depression reduction in both groups. This led to a medium overall ES, −0.52 ES (95% CI −0.98, −0.06; *P* < .05) that met both the 1 SEM and 0.5 SD criteria, favoring overall depression reduction in the yoga group relative to the supportive therapy control. Within the Chandwani et al. study [10], small clinically significant differences in the yoga group, −0.48 ES (95% CI −0.86, −0.09; *P* < .05), and small clinically significant differences in the waitlist control group, −0.32 ES (95% CI −0.67, 0.03; *P* = NS), also indicated significant depression reduction in both groups. However, there were no overall clinically significant differences in depression reduction between yoga and waitlist control groups. Within the Cohen et al. [11] study there were no clinically significant differences in depression reduction pre-post or between groups. Within single group designs, ES ranged from small, −0.36 ES (95% CI −0.66, −0.05; *P* < .05) meeting the 1 SEM criteria [19], to large, −1.00 ES (95% CI −1.69, −0.32; *P* < .01) meeting both the 1 SEM and 0.5 SD[18]. In the case of Speed-Andrews et al. [22] no clinically significant differences in depression were indicated pre-post yoga intervention.

#### 3.3.2. Anxiety

Within the control designs, Vadiraja et al. [17] indicated moderate clinically significant differences, with an overall between-groups ES of −0.51 (95% CI −0.97, −0.05; *P* < .05) that met the 1 SEM criteria. Chandwani et al.[10] indicated moderate clinically significant differences within the yoga group, −0.63 ES (95% CI −1.04, −0.23; *P* < .01) and small clinically significant differences in the waitlist control group, ES −0.20 (95% CI −0.55, 0.15; *P* = NS), indicating anxiety reduction in both groups. Overall, small clinically significant differences between yoga and waitlist control, with an ES of −0.46 (95% CI −0.98, 0.05; *P* = NS) that met both the 1 SEM and 0.5 SD criteria, suggest relative overall anxiety reduction in the yoga group relative to the waitlist control. Interestingly, within the Cohen et al. study [11], small clinically significant differences, with an ES of −0.30 (95% CI −0.80, 0.21; *P* = NS) that met the 1 SEM criteria, indicate significant overall anxiety reduction in the waitlist control versus the yoga group. Within single group designs Ülger and Yağlı [23] demonstrated large clinically significant reductions in anxiety, −1.51 ES (95% CI −2.14, −0.88; *P* < .001) that met both the 1 SEM and 0.5 SD criteria. Danhauer et al. [19] indicated small clinically significant reductions in anxiety, with an ES of −0.22 (95% CI −0.52, 0.08; *P* = NS) that met the 1 SEM criteria. No clinically significant differences were reported in Speed-Andrews et al. [22].

#### 3.3.3. Positive Affect

Danhauer et al. [13]demonstrated a small clinically significant effect of 0.45 ES (95% CI −0.29, 1.19; *P* = NS) that met the 1 SEM criteria between yoga and waitlist control groups. Vadiraja et al. [16] reported a moderate clinically significant effect pre-post yoga intervention of 0.52 ES (95% CI 0.20, 0.84). However, although statistically significant (*P* < .001), the between-group difference met neither the 1 SEM nor 0.5 SD criteria. Within the single group designs, there were no significant differences in positive affect pre-post yoga intervention.

#### 3.3.4. Negative Affect

Within the Danhauer et al. study [13], moderate clinically significant differences between groups, −0.73 ES (95% CI −1.49, 0.03; *P* = NS) meeting both the 1 SEM and 0.5 SD criteria, indicated significant overall reduction in negative affect in the yoga group. Within the Vadiraja et al. study [16], large clinically significant differences within the yoga group, −0.86, ES (95% CI −1.21, −0.51; *P* < .001) and small differences within the supportive therapy group, −0.33 ES (95% CI −0.67, 0.01; *P* = NS) suggested there was a clinically significant reduction of negative affect in both groups. However, moderate clinically significant differences between groups, −0.57 ES (95% CI −1.03, −0.11; *P* < .05), that met both the 1 SEM and 0.5 SD criteria, suggest a significant overall reduction of negative effect in the yoga group relative to the supportive therapy control group. In the single group study designs, Danhauer et al. [19] met neither the 1 SEM nor 0.5 SD criteria but did exhibit a statistically significant (*P* < .05) ES of −0.35 (95% CI −0.65, −0.04).

#### 3.3.5. Spiritual Well-Being

In Danhauer et al. [13] moderate clinically significant differences, 0.56 ES (95% CI −0.19, 1.30; *P* = NS) meeting both the 1 SEM and 0.5 SD criteria, indicated an increase in spiritual well-being in the yoga versus waitlist control group. Within the Moadel et al. study [15] there were no clinically significant differences in spiritual well-being pre-post or between groups. Within the single group designs moderate clinically significant differences, 0.60 ES (95% CI 0.16, 1.04; *P* < .01) were reported pre-post yoga intervention [20]. There were no clinically significant differences in spiritual well-being pre-post yoga intervention in Danhauer et al. [19].

### 3.4. Symptom Outcomes

#### 3.4.1. Fatigue

Within the control group designs [10, 11, 13–15] clinically significant differences between the yoga intervention and control groups ranged from small ES, −0.17 ES (95% CI −0.68, 0.34; *P* = NS), meeting the 1 SEM criteria [10] to medium ES, 0.71 ES (95% CI −0.04, 1.47; *P* = NS), meeting both the 1 SEM and 0.5 SD criteria [13]. In the case of Cohen et al. [11] there were no clinically significant differences in fatigue pre-post or between yoga and control groups. Within the single group designs, effect sizes ranged from small, 0.22 ES (95% CI −0.08, 0.52; *P* = NS) meeting the 1 SEM criteria [19], to very large, −2.34 ES (95% CI −3.47 to −1.22; *P* < .001) meeting both the 1 SEM and 0.5 criteria [18].

#### 3.4.2. Sleep

In the case of Danhauer et al. [13] despite small clinically significant differences pre-post, ES −0.46 ES (95% CI −1.00, 0.08; *P* = NS) there were no overall clinically significant differences in sleep between the yoga and waitlist control groups. There were no clinically significant differences in sleep pre-post or between groups in the Chandwani or Cohen studies [10, 11]. In the single group designs, there was a large effect, −0.88 ES (95% CI −1.38, −0.38; *P* < .001) meeting both 1 SEM and 0.5 SD requirements, for sleep pre-post yoga intervention [23]. In the case of Bower et al.[18] there were no clinically significant differences in sleep pre-post yoga intervention.

### 3.5. Clinical Significance Criteria

#### 3.5.1. Consistency between SEM and SD

Within the control group designs, the correlation between the 1 SEM and 0.5 SD criteria for HRQL indices was *r* = .92, *P* < .01 for the treatment group and *r* = .93, *P* < .01 for the control group. For the single group designs the correlation between 1 SEM and 0.5 SD was *r* = .92, *P* < .01, indicating these criteria are highly correlated. For psychosocial indices within the control group designs, the overall correlation between the 1 SEM and 0.5 SD criteria was *r* = .74, *P* < .01 for the treatment group and *r* = .83, *P* < .01 for the control group. For the single group designs the correlation between 1 SEM and 0.5 SD was *r* = .80, *P* < .01, indicating these criteria are also highly correlated. Similarly, for the symptom indices, within the control group designs, the overall correlation between the 1 SEM and 0.5 SD criteria was *r* = .91, *P* < .01 for the treatment group and *r* = .91, *P* < .01 for the control group. Finally, for the single group designs the correlation between 1 SEM and 0.5 SD was *r* = .97, *P* < .05, indicating these criteria are highly correlated.

## 4. Discussion

The yoga and cancer literature is rapidly growing. This literature is characterized by studies published with small sample sizes and variability in the type and length of interventions, populations studied (e.g., cancer type, time during the treatment continuum), and measures used to assess the patient-reported outcomes of interest. Thus, to accurately characterize the results of the current literature in this area, reporting multiple indicators of clinical significance is of great value [38]. Studies evaluated for clinical significance show some consistency across this research, demonstrating the positive impact of yoga on quality of life, psychosocial, and, to a lesser degree, physical symptom indices.

 Since this area of research is in its infancy, it is useful to utilize multiple criteria of clinical significance to explore the clinical efficacy of results from each study. We recommend the use of not only ES and CIs, but also validated metrics such as the 1 SEM and 0.5 SD criteria. A cursory look at our summary tables would indicate that to examine any of these studies by one criterion alone, particularly in the case of *P*-values, much information and insight into the results would be lost. Based on our current paper, we see emerging beneficial findings from yoga interventions in the areas of HRQL, including overall HRQL and its mental and emotional domains, and in psychosocial measures in the areas of anxiety, depression, negative affect, and spiritual well-being. There may also be some preliminary evidence for yoga's role in fatigue. Findings for the role of yoga in physical, functional, and social domains of HRQL remain far more inconclusive, as is the role of yoga in positive affect and sleep indices for cancer survivors. Thus, considering clinical significance indicates stronger support than previous reviews of the literature for the preliminary efficacy of yoga for improving overall HRQOL and its mental and emotional domains, in addition to psychosocial outcomes [6, 7].

The present paper highlights that yoga for cancer survivors results in a number of clinically significant improvements in select patient-reported outcomes. Specifically, multiple criteria, including a mean difference from pre- to postintervention greater than or equal to 1 SEM and/or 0.5 SD, and the respective ES and CI, were met for quality of life and psychosocial outcomes (e.g., anxiety, depression, positive and negative effect, and spiritual well-being), and for some limited symptom outcomes (e.g., fatigue, sleep). These results suggest that indices vary in their sensitivity/conservatism for reporting clinical significance. For example, in cases where research participants patient-reported outcome pre-scores are subclinical or it has been established that smaller changes in patient-reported outcomes would be beneficial (e.g., palliation), using the mean difference criterion of greater than 1 SEM may be a more pragmatic index of clinically significant change. In addition, researchers may want to employ the respective smaller ES with the SEM when smaller changes would be considered meaningful.

### 4.1. Baseline Cut-offs Reported in Literature

One consideration for the discrepancies between clinical significance and statistical significance found for HRQOL and psychosocial patient-reported outcomes may be participant baseline characteristics. Baseline health status, as reflected in patient-reported outcome scores, has been shown to affect the overall responsiveness of an instrument [39]. For example, if participants are several months posttreatment, they may report preintervention scores that are relatively high (positive). Thus, there is less “room” for improvement and a ceiling effect is encountered on a given outcome. The patient cannot score any higher and so a given level of change necessary for clinical significance is not obtained [28]. Therefore, improvements in patient-reported outcomes must be contextualized within participant baselines scores.

While beyond the scope of this manuscript, the baseline values of all HRQL, psychosocial, and symptom measures varied for each study in comparison to established clinical cutoffs, where available. Clinical cut-off values are established to distinguish between people who have a clinically important level of the construct and to enable comparison between studies. Thus studies that had more room for improvement at baseline, which can be judged in comparison to clinical cut-off values, may have resulted in higher levels of clinical significance.

### 4.2. MIDs Reported in Literature

In addition to the 1 SEM, 0.5 SD, and ES measures reported in our results, for some instruments, MIDs currently exist in the literature. Thus it may also be of value to compare current results to literature-reported MIDs. Utilizing a combination of distribution-based methods and MIDs reported in the literature, as they are available, allows for unique insights into both the interventions and study members. While literature-based MIDs may allow for additional ease in comparing changes across studies, they are developed based on a large cross-section of studies and may not always be appropriate given any one study [38].

### 4.3. Limitations and Future Research

The current paper has some limitations. First, it is important to note calculations of clinical significance do not account for elements that can be controlled by the original study design. Differences between the studies—type of yoga, duration, assessment periods, number of assessments, type of control group (waitlist versus active comparison control), sample size, and cancer type—are all issues that should be considered in the interpretation of the results. For example, some studies may show clinically significant changes as a function of a longer duration (i.e., a time effect improvement in patient-reported outcomes) rather than a direct benefit of yoga. In addition, small sample sizes may also miss important clinical differences and mask significant improvement following an intervention. In contrast, studies with large sample sizes may produce significant findings that are clinically meaningless [40]. Future studies will be strengthened by the recruitment of larger, more homogenous samples to minimize this variability and lead to more consistent results. In addition, the research clearly has relied primarily on participation from breast cancer survivors, and minimal published work on yoga in other groups of cancer survivors exists to date.

The relative dearth of symptom-focused patient-reported outcomes illustrates a gap in the evaluation of the potential physical effects of yoga for cancer survivors. Whereas it is certainly important that yoga consistently demonstrates a positive influence on quality of life and psychosocial outcomes, research evaluation may be missing an important component of the potential benefits that may be gained in symptom alleviation, be it fatigue, sleep disturbance, or pain. Future research that focuses more in-depth on assessment of symptom patient-reported outcomes, in addition to fatigue and sleep, is warranted.

Comparing yoga to other types of interventions, either physical activity or psychosocial, may elucidate comparable effects or unique outcomes of yoga on patient-reported outcomes in cancer survivors. Notably, yoga was not originally intended to target specific outcomes. Thus, choosing which of the many yoga practices to implement (e.g., movement, breath, and meditation) and, based in part on these decisions, which psychophysiological outcomes to assess will be an ongoing challenge in the attempt to explore this holistic ancient practice with contemporary methods of scientific exploration [5].

Within this paper a total of 18 different instruments were used to assess patient-reported outcomes, including six instruments for HRQL alone. Recent research has advocated for a more coordinated use of measures to increase validity of constructs measured as well as to ensure the reliability of comparisons [41]. In this capacity, recent work by the Patient Reported Outcomes Measurement Information System (PROMIS) cooperative group provides new direction in collectively and parsimoniously measuring these phenomena [42]. Independent of the PROMIS initiative, the literature on yoga for cancer survivors is beginning to use some of the same measures within multiple interventions. This practice of consistent use of measures is valuable for furthering the evidence on yoga for cancer survivors. For example, recent studies have found clinically significant changes using the FACT-G and subscales, FACT-F, and FACIT-Sp [13, 20, 22]. Thus, we now have the benefit of inferring that it was not likely the measure alone, but perhaps other intervention-dependent factors, that influenced the results. In this capacity, we also strongly recommend the use of disease-specific instruments in future research.

Identifying mechanisms that explain how yoga leads to clinically significant outcomes is another next step in understanding the ability of yoga to target specific desired outcomes. Little research attention has been paid to the psychophysiological mechanisms by which benefits are accrued via yoga practice. In this capacity, there have been recommendations to integrate patient-reported outcomes with biomedical endpoints as a means of better describing the complexity of these measures [43]. Before yoga can be broadly applied within oncology, both a strong theoretical understanding of how yoga practice causes change and carefully designed and executed research that convincingly evaluates not only the efficacy of yoga in clinical settings but also posits potential mechanisms of action underlying these interventions are required [44].

Finally, since the timing of the assessment of a patient-reported outcome may influence clinical significance, it is important for future studies of yoga to include measures that look at change before and after a single yoga practice session. Assessing these more acute effects of yoga would allow for documentation of the potential immediate impact of yoga on outcomes of interest [45].

## 5. Conclusion

There is a need across the research literature to assess a variety of indices that speak to a given study's clinical significance, a metric relevant to researchers, clinicians, and patients alike. The current analysis supports preliminary evidence of the clinical significance of yoga for improving quality of life, psychosocial, and limited symptom outcomes in cancer survivors. It behooves researchers to take these preliminary findings as a starting point to begin to explore, report and revise these purported indices of clinical significance further [30]. Identifying the clinical significance in studies of yoga for cancer survivors adds further description to the existing literature and highlights the promising benefits of a yoga intervention. While this study focused specifically on one type of intervention in a defined group (i.e., cancer survivors), there is the broader issue of the need to examine and present clinical significance data for other types of intervention studies in order to ensure that “significant” findings are truly meaningful to people, impacting various health outcomes and behaviors that are important to them.

## Figures and Tables

**Table 1a:** (a)

Author	Sample	Cancer Type and Treatment	Yoga Intervention	Control Group	Outcome Measures Reported in Current Review
Chandwani et al., 2010 [10]	Treatment: T1 = 30, T2 = 27 Control: T1 = 31, T2 = 31 Age range: 31–68 Mean age = 53	Breast (Stages 0–3) Chemotherapy (*n* = 47) Radiation therapy (*n* = 61)	60-minute classes twice weekly for 6 weeks Type: Vivekananda Yoga	Wait-list control	Quality of Life -SF-36 Psychosocial -CES-D, STAI Symptom -BFI, PSQI

Cohen et al., 2004 [11]	Treatment: T1 = 19, T2 = 16 Control: T1 = 19, T2 = 14 Age range: NR Mean age = 51	Lymphoma (Stages I–IV) Previous 12 months receiving chemotherapy	Weekly classes for 7 weeks (class length not reported) Type: Tibetan Yoga	Wait-list control	Psychosocial -CES-D, STAI Symptom -BFI, PSQI

Culos-Reed et al., 2006 [12]	Treatment: T1 = 20, T2 = 18 Control: T1 = 18, T2 = 18 Age range: NR Mean age = 51	Breast (*n* = 32), Other (*n* = 6) ≥3 months post-treatment	75 minute weekly classes for 7 weeks Type: Iyengar Yoga	Wait-list control	Quality of Life -EORTC QLQ-C30 Psychosocial -POMS

Danhauer et al., 2009 [13]	Treatment:T1 = 22, T2 = 13 Control: T1 = 22, T2 = 14 Age range: 38–79 Mean age = 55	Breast (Stages I–IV) Radiation therapy (*n* = 9) Chemotherapy (*n* = 11)	75-minute weekly classes for 10 weeks Type: Integral Yoga	Wait-list control	Quality of Life -FACT-B, SF-12 Psychosocial -CES-D, PANAS, FACIT-Sp Symptom -FACT-F, PSQI

Littman et al., 2011 [14]	Treatment: T1 = 32, T2 = 27 Control: T1 = 31, T2 = 27 Age range: 33–74 Mean age = 60	Breast (Stages 0–III) >3 months post-hemotherapy and/or post-radiation	75-minute classes five times per week for 26 weeks (combination in-facility and home-based practice) Type: Viniyoga	Wait-list control	Quality of Life -FACT-G Symptom -FACT-F

Moadel et al., 2007 [15]	Treatment: T1 = 45, T2 = 45 Control: T1 = 26, T2 = 26 Age range: 28–75 Mean age = 54	Breast (Stages I–III) Surgery (*n* = 95) Radiation Therapy (*n* = 10)Chemotherapy (*n* = 27)Anti-estrogen Therapy (*n* = 30)	90 minute weekly classes for 12 weeks. Home practice encouraged Type: Hatha Yoga	Wait-list control	Quality of Life -FACT-G Psychosocial -FACIT-Sp Symptom -FACT-F

Vadiraja et al., 2009a and b [16, 17]	Treatment: T1 = 44, T2 = 42 Control: T1 = 44, T2 = 33 Age range: 30–70 Mean age = 46	Breast (Stages II-III) Radiation therapy (*n* = 88)	Minimum of 3 in-person 60-minute individual yoga sessions per week for 6 weeks. Home practice encouraged. Type: Integrated Yoga Program	3-4 sessions of brief supportive therapy w/education	Quality of Life -EORTC QoL C30 Psychosocial -PANAS, HADS

**Table 1b:** (b)

Author	Sample	Cancer Type and Treatment	Yoga Intervention	Outcome Measures Reported in Current Review
Bower et al., 2011 [18]	T1 = 12, T2 = 11 Age range: 46–65 Mean age = 54	Breast (Stages 0–2) All had completed radiation therapy or radiation therapy + chemotherapy	90-minute classes twice weekly for 12 weeks Type: Iyengar Yoga	Quality of Life -SF-36 Psychosocial -BDI-II Symptom -PSQI, FSI

Danhauer et al., 2008 [19]	T1 = 51, T2 = 43 Age range: 34–82 Mean age = 59	Ovarian (*n* = 37) Breast (*n* = 14) Radiation therapy (*n* = 5) Chemotherapy (*n* = 29)	75-minute weekly classes for 10 weeks Type: Integral Yoga	Quality of Life -FACT-G, SF-12 Psychosocial -CES-D, FACIT-Sp, PANAS, STAI Symptom -FACT-F

Duncan et al., 2008 [20]	T1 = 24, T2 = 22 Age range: NR Mean age = 49	Breast (42%) Gynecologic (16.6%) Lymphoma (12.5%) Currently on or <6 months post treatment	90 minute weekly classes for 10 weeks Type: Iyengar Yoga	Quality of Life -FACT-G Psychosocial -FACIT-Sp

Galantino et al., 2011 [21]	T1 = 10, T2 = 10 Age range: 50–71 Median age = 58	Breast (Stages I–III) >4 weeks post-chemotherapy and/or post-radiation, all women taking aromatase inhibitors	90-minute classes twice weekly for 8 weeks, home practice encouraged Type: Iyengar-inspired	Quality of Life -FACT-B

Speed-Andrews et al., 2010 [22]	T1 = 17, T2 = 17 Age range: not reported Mean age = 54	Breast (Stages I–III) Surgery (*n* = 23) Radiation therapy (*n* = 19) Chemotherapy (*n* = 18) Hormone therapy (*n* = 10)	Two different sessions; 90 minute classes twice weekly for 6 weeks and 90 minute classes 22 times over 12 weeks Type: Iyengar Yoga	Quality of Life -FACT-B, SF-36 Psychosocial -CES-D, STAI

Ülger and Yağlı, 2010 [23]	T1 = 20, T2 = 20 Age range: 30–50 Mean age = 41	Breast >6 months post-chemotherapy	60 minute weekly classes for 8 weeks Type: Not specified (“Classical Yoga”)	Quality of Life -NHP Psychosocial -STAI

Abbreviations: T1: baseline; T2: follow-up; SF-36 and SF-12: Medical Outcomes Study Short-Form Health Survey; EORTC QLQ C30: European Organization for Research and Treatment of Cancer-Quality of Life C30; FACT-B: Functional Assessment of Cancer Therapy—Breast; FACT-G: Functional Assessment of Cancer Therapy—General; NHP: Nottingham Health Profile; BDI: Beck Depression Inventory; CES-D 20: Center for Epidemiologic Studies Depression Scale—20 Item; STAI: State/Trait Anxiety Inventory; POMS: Profile of Mood States; PANAS: Positive and Negative Affect Schedule; FACIT-Sp: Functional Assessment of Chronic Illness Therapy-Spiritual Well-Being; HADS: Hospital Anxiety and Depression Scale; BFI: Brief Fatigue Inventory; PSQI: Pittsburgh Sleep Quality Index; FSI: Fatigue Symptom Inventory; FACT-F: Functional Assessment of Cancer Therapy-Fatigue. NR: Not Reported.

**Table 2a:** (a)

Study	Outcome measure	Internal consistency (Cronbach *α*)	SEM (Baseline SD × *√*1 − Cronbach *α*)		Baseline 0.5 SD		Baseline Mean (SD)		Within-Groups Mean Difference (95% CI)		Within-Groups Effect Size—Hedges' g (95% CI)		Between Groups Mean Difference (95% CI)	Between Groups Effect size—Hedges' g (95% CI)
			Yoga	Control	Yoga	Control	Yoga	Control	Yoga	Control	Yoga	Control
Chandwani et al., 2010 [10]	SF36 PCS						41.50	41.8	1.60	−2.80	0.18	−0.27	4.40	0.46
	(Physical)	.90	2.60	3.40	4.10	5.30	(8.20)	(10.6)	[−1.74, 4.94]	[−6.35, 0.75]	[−0.19, 0.54]	[−0.62, 0.08]	[−0.52, 9.32]^c^	[−0.06, 0.97]
	SF36 MCS						47.80	48.0	1.90	1.90	0.17	0.18	0.00	0.00
	(Mental)	.90	3.80	3.50	6.00	5.60	(12.00)	(11.1)	[−2.16, 5.96]	[−1.76, 5.56]	[−0.19, 0.54]	[−0.17, 0.52]	[−5.45, 5.45]	[−0.51, 0.51]

Culos-Reed et al., 2006 [12]	EORTC-QoL						64.58	62.04	13.66	0.46	0.56	0.03	13.20	0.67
	(Overall)	.89	8.50	4.26	12.82	6.43	(25.63)	(12.85)	[2.85, 24.47]^ab^	[−6.16, 7.08]	[0.08, 1.04]*	[−0.41, 0.47]	[0.52, 25.88]^cd^	[0.01, 1.32]*
	EORTC-Emo						79.58	75.00	4.22	−4.17	0.23	−0.25	8.39	0.48
	(Emotional)	.80	9.01	6.64	10.07	7.43	(20.14)	(14.85)	[−4.05, 12.49]	[−11.57, 3.23]	[−0.22, 0.67]	[−0.70, 0.20]	[−2.71, 19.49]^cd^	[−0.16, 1.13]

Danhauer et al., 2009 [13]	FACT-B						104.90	101.1	9.90	−2.70	0.48	−0.09	12.6	0.49
	(Overall)	.90	6.30	7.70	10.00	12.20	(19.90)	(24.4)	[−0.71, 20.51]^a^	[−17.80, 12.40]	[−0.06, 1.02]	[−0.58, 0.41]	[−6.12, 31.32]^cd^	[−0.25, 1.24]
	FACT-SWB						23.30	21.4	−0.20	−1.00	−0.04	−0.15	0.80	0.14
	(Social)	.69	2.60	3.00	2.60	2.70	(4.70)	(5.4)	[−2.84, 2.44]	[−4.26, 2.26]	[−0.55, 0.47]	[−0.65, 0.35]	[−3.43, 5.03]	[−0.60, 0.87]
	FACT-FWB						18.70	18.0	3.20	−0.60	0.57	−0.08	3.80	0.58
	(Functional)	.86	2.60	3.10	2.90	3.50	(5.70)	(6.9)	[0.33, 6.07]^ab^	[−4.38, 3.18]	[0.01, 1.12]*	[−0.57, 0.42]	[−1.00, 8.60]^cd^	[−0.17, 1.34]
	FACT-EWB						18.10	18.5	2.70	−0.30	0.70	−0.05	3.00	0.61
	(Emotional)	.69	2.00	2.70	2.00	2.60	(3.90)	(5.2)	[0.74, 4.66]^ab^	[−3.29, 2.69]	[0.13, 1.28]*	[−0.54, 0.44]	[−0.63, 6.63]^cd^	[−0.15, 1.35]
	FACT-PWB						19.70	20.7	2.80	0.40	0.36	0.07	2.40	0.36
	(Physical)	.81	3.00	2.20	3.50	2.60	(7.00)	(5.1)	[−1.18, 6.78]	[−2.44, 3.24]	[−0.17, 0.89]	[−0.42, 0.56]	[−2.44, 7.24]^c^	[−0.38, 1.10]
	SF-12 PCS						42.70	40.6	2.10	2.10	0.16	0.18	0.00	0.00
	(Physical)	.86	4.50	3.80	6.1 0	5.10	(12.10)	(10.1)	[−4.56, 8.76]	[−3.68, 7.88]	[−0.35, 0.67]	[−0.32, 0.68]	[−8.79, 8.79]	[−0.73.0.73]
	SF-12 MCS						43.40	49.9	8.80	−2.40	0.93	−0.18	11.20	1.00
	(Mental)	.81	4.40	4.50	5.10	5.10	(10.10)	(10.2)	[3.97, 13.63]^ab^	[−8.90, 4.10]	[0.30, 1.55]**	[−0.68, 0.31]	[3.01, 19.39]^cd^	[0.22, 1.78]**

Littman et al., 2011 [14]	FACT-B						89.00	87.8	1.30	−0.10	0.12	−0.01	1.40	0.11
	(Overall)	.90	2.97	4.49	4.70	7.10	(9.40)	(14.2)	[−2.58, 5.18]	[−5.61, 5.41]	[−0.24, 0.49]	[−0.37, 0.36]	[−5.34, 8.14]	[−0.42, 0.64]
	FACT-SWB						21.70	21.7	0.40	−0.80	0.08	−0.14	1.20	0.22
	(Social)	.69	2.84	3.01	2.60	2.70	(5.10)	(5.4)	[−1.51, 2.31]	[−2.96, 1.40]	[−0.29, 0.44]	[−0.50, 0.23]	[−1.68, 4.08]	[−0.31, 0.75]
	FACT-FWB						22.70	21.6	−0.10	0.10	−0.03	0.02	0.20	0.05
	(Functional)	.86	1.23	1.46	1.70	2.60	(3.30)	(5.1)	[−1.47, 1.27]	[−1.75, 1.95]	[−0.39, 0.34]	[−0.35, 0.39]	[−2.11, 2.51]	[−0.50, 0.57]
	FACT-EWB						19.80	20.3	0.50	0.50	0.14	0.17	0.00	0.00
	(Emotional)	.69	1.56	1.45	1.40	1.30	(2.80)	(2.6)	[−0.84, 1.84]	[−0.59, 1.59]	[−0.23, 0.51]	[−0.20, 0.54]	[−1.73, 1.73]	[−0.53, −0.53]
	FACT-PWB						24.70	24.2	0.70	0.10	0.33	0.02	0.60	0.18
	(Physical)	.81	1.00	1.7 0	1.20	2.00	(2.30)	(3.9)	[−0.08, 1.48]	[−1.47, 1.67]	[−0.05, 0.71]	[−0.34, 0.39]	[−1.16, 2.36]	[−0.35, 0.71]

Moadel et al., 2007 [15]	FACT-G						76.53	77.54	1.54	−7.16	0.09	−0.30	8.70	0.43
	(Overall)	.89	5.96	8.11	8.99	12.23	(17.98)	(24.45)	[−3.60, 6.68]	[−16.25, 1.93]	[−0.20, 0.37]	[−0.68, 0.09]	[−0.96, 18.36]^c^	[−0.05, 0.91]
	FACT-SWB						20.98	22.12	−0.30	−3.95	−0.05	−0.60	3.65	0.60
	(Social)	.69	3.47	3.46	3.12	3.11	(6.23)	(6.22)	[−1.99, 1.39]	[−6.41, −1.49]^ab^	[−0.34, 0.24]	[−1.00, −0.19]**	[0.75, 6.56]^cd^	[0.11, 1.09]*
	FACT-FWB						18.33	18.19	−0.16	−1.98	−0.02	−0.26	1.82	0.26
	(Functional)	.80	2.85	3.45	3.19	3.86	(6.38)	(7.72)	[−2.08, 1.76]	[−4.88, 0.92]	[−0.31, 0.26]	[−0.63, 0.12]	[−1.53, 5.17]	[−0.22, 0.74]
	FACT-EWB						16.36	16.50	1.83	−0.41	0.40	−0.07	2.24	0.44
	(Emotional)	.74	2.43	3.03	2.39	2.97	(4.77)	(5.94)	[0.51, 3.14]**	[−2.65, 1.83]	[0.10, 0.70]**	[0.44, 0.31]	[−0.19, 4.67]	[−0.05, 0.92]
	FACT-PWB						20.87	20.73	0.16	−0.82	0.03	−0.12	0.98	0.16
	(Physical)	.82	2.24	2.98	2.64	3.52	(5.28)	(7.03)	[−1.51, 1.83]	[−3.39, 1.75]	[−0.26, 0.32]	[−0.49, 0.26]	[−1.96, 3.91]	[−0.32, 0.64]

Vadiraja et al., 2009 [16]	EORTC-Phys						73.20	62.72	0.06	6.24	0.00	0.20	6.18	0.23
	(Physical)	.71	12.49	16.68	11.60	15.49	(23.20)	(30.98)	[−7.30, 7.42]	[−4.19, 16.66]	[−0.29, 0.30]	[−0.14, 0.54]	[−6.24, 18.60]	[−0.23, 0.68]
	EORTC-Role						72.72	71.59	7.16	1.26	0.20	0.03	5.90	0.16
	(Role)	.52	24.15	25.22	17.43	18.20	(34.86)	(36.40)	[−3.32, 17.64]	[−11.81, 14.33]	[−0.10, 0.50]	[−0.30, 0.37]	[−10.64, 22.44]	[−0.29, 0.61]
	EORTC-Emo (Emotional)						56.45	51.58	18.67	7.65	0.89	0.36	11.02	0.53
		.80	8.84	7.80	9.89	8.72	(19.77)	(17.44)	[12.47, 24.87]^ab^	[0.48, 14.82]	[0.54, 1.25]***	[−0.01, 0.70]*	[1.57, 20.47]^cd^	[0.07, 0.99]*
	EORTC-Cog						85.29	82.67	5.28	−1.90	0.30	−0.08	7.18	0.36
	(Cognitive)	.73	9.35	10.97	9.00	10.56	(18.00)	(21.12)	[0.13, 10.43]	[−9.66, 5.86]	[0.00, 0.61]*	[−0.42, 0.25]	[−1.83, 16.19]	[−0.10, 0.82]
	EORTC-Soc						52.82	52.41	2.14	−2.48	0.08	−0.10	4.62	0.18
	(Social)	.77	12.73	11.72	13.28	12.22	(26.55)	(24.43)	[−5.53, 9.81]	[−10.78, 5.82]	[−0.22, 0.38]	[−0.43, 0.23]	[−6.74, 15.98]	[−0.27, 0.64]

**Table 2b:** (b)

Study	Outcome measure	Internal consistency (Cronbach *α*)	SEM (Baseline SD × *√*1 − Cronbach *α*)		Baseline 0.5 SD		Baseline Mean (SD)		Within-Groups Mean Difference (95% CI)		Within-Groups Effect Size—Hedges' g (95% CI)		Between Groups Mean Difference (95% CI)	Between Groups Effect size—Hedges' g (95% CI)
			Yoga	Control	Yoga	Control	Yoga	Control	Yoga	Control	Yoga	Control
Chandwani et al., 2010 [10]	CES-D 20						11.60	10.50	−5.00	−3.50	−0.48	−0.32	−1.50	−0.14
	(Depression)	.87	3.35	2.42	4.65	3.35	(9.30)	(6.70)	[−8.85, −1.15]^ab^	[−7.23, 0.24]^a^	[−0.86, −0.09]*	[−0.67, 0.03]	[−6.87, 3.87]	[−0.65, 0.37]
	STAI-State						36.30	32.70	−8.30	−2.50	−0.63	−0.20	−5.80	−0.46
	(Anxiety)	.94	3.36	2.45	6.85	5.00	(13.70)	(10.00)	[−13.09, −3.51]^ab^	[−6.75, 1.75]^a^	[−1.04, −0.23]**	[−0.55, 0.15]	[−12.18, 0.58]^cd^	[−0.98, 0.05]

Cohen et al., 2004 [11]	CES-D 20						10.20	9.60	−1.20	.10	−0.12	0.01	−1.30	−0.15
	(Depression)	.93	2.91	2.27	5.50	4.29	(11.00)	(8.57)	[−5.91, 3.51]	[−3.79, 4.00]	[−0.58, 0.35]	[−0.48, 0.51]	[−7.52, 4.92]	[−0.85, 0.55]
	STAI-State						34.30	37.80	−0.20	−4.00	−0.02	−0.30	−3.80	−0.31
	(Anxiety)	.95	2.75	3.26	6.15	7.30	(12.30)	(14.60)	[−5.54, 5.14]	[−10.65, 2.65]^a^	[−0.48, 0.45]	[−0.80, 0.21]	[−12.24, 4.64]^c^	[−1.02, 0.39]

Culos-Reed et al., 2006 [12]	POMS-D						4.70	5.44	−2.48	0.06	−0.34	0.01	−2.54	−0.39
	(Depression)	.95	1.76	1.14	3.93	2.55	(7.86)	(5.10)	[−5.68, 0.72]^a^	[−2.54, 2.66]	[−0.80, 0.11]	[−0.43, 0.45]	[−6.66, 1.58]^c^	[−1.04, 0.25]

Danhauer et al., 2009 [13]	CES-D 20						16.30	16.60	−8.20	1.20	−0.82	0.07	−9.40	−0.69
	(Depression)	.93	2.57	3.89	4.85	7.35	(9.70)	(14.70)	[−13.27, −3.17]^ab^	[−7.14, 9.54]	[−1.42, −0.22]**	[−0.42, 0.57]	[−19.34, 0.54]^cd^	[−1.45, 0.06]
	PANAS-P						32.70	30.60	5.50	1.20	0.64	0.11	4.30	0.45
	(Positive Affect)	.87	3.17	3.53	4.40	4.90	(8.80)	(9.80)	[1.16, 9.84]^ab^	[−4.21, 6.61]	[0.08, 1.21]*	[−0.39, 0.60]	[−2.71, 11.31]^c^	[−0.29, 1.19]
	PANAS-N						19.00	18.50	−5.00	1.40	−0.65	0.14	−6.40	−0.73
	(Negative Affect)	.87	2.99	3.39	4.15	4.70	(8.30)	(9.40)	[−8.91, −1.09]^ab^	[−3.63, 6.43]	[−1.22, 0.08]*	[−0.36, 0.63]	[−12.84, 0.04]^cd^	[−1.49, 0.03]
	FACIT-Sp						23.30	23.20	2.70	−1.70	0.38	−0.19	4.40	0.56
	(Spiritual Well-Being)	.87	2.38	2.60	3.30	3.60	(6.60)	(7.20)	[−0.92, 6.31]^a^	[−6.16, 2.76]	[−0.15, 0.91]	[−0.69, 0.31]	[−1.40, 10.20]^cd^	[−0.19, 1.30]

Moadel et al., 2007 [15]	FACIT-Sp						37.87	34.58	−1.02	−1.83	−0.11	−0.15	0.81	0.08
	(Spiritual Well-Being)	.87	3.33	3.99	4.62	5.53	(9.24)	(11.06)	[−3.59, 1.55]	[−6.49, 2.83]	[−0.40, 0.17]	[−0.52, 0.23]	[−4.08, 5.70]	[−0.40, 0.56]

Vadiraja et al., 2009 [16]	PANAS-P						24.05	21.81	3.80	1.52	0.52	0.19	2.28	0.30
	(Positive Affect)	.87	2.62	2.66	3.64	3.69	(7.28)	(7.37)	[1.62, 5.98]^ab^	[−1.17, 4.21]	[0.20, 0.84]***	[−0.15, 0.53]	[−1.14, 5.70]	[−0.15, 0.76]
	PANAS-N						22.15	25.22	−9.24	−3.37	−0.86	−0.33	−5.87	−0.57
	(Negative Affect)	.87	3.82	3.18	5.30	4.41	(10.60)	(8.82)	[−12.41, −6.074]^ab^	[−6.78, 0.04]^a^	[−1.21, −0.51]***	[−0.67, 0.01]	[−10.56, −1.18]^cd^	[−1.03, −0.11]*

Vadiraja et al., 2009b [17]	HADS-A						8.05	9.35	−3.17	−1.23	−0.86	−0.31	−1.94	−0.51
	(Anxiety)	.79	1.77	1.82	1.94	1.99	(3.87)	(3.98)	[−4.27, −2.07]^ab^	[−2.56, 0.10]	[−1.21, −0.51]***	[−0.65, 0.03]	[−3.65, −0.23]^c^	[−0.97, −0.05]*
	HADS-D						7.57	8.00	−3.43	−1.47	−0.89	−0.40	−1.96	−0.52
	(Depression)	.87	1.45	1.25	2.01	1.74	(4.02)	(3.47)	[−4.57, −2.29]^ab^	[−2.71, −0.23]^a^	[−1.25, −0.54]***	[−0.74, −0.05]*	[−3.65.−0.27]^cd^	[−0.98, −0.06]*

**Table 2c:** (c)

Study	Outcome measure	Internal consistency (Cronbach *α*)	SEM (Baseline SD × *√*1 − Cronbach's *α*)		Baseline SD 0.5		Baseline Mean (SD)		Within-Groups Mean Difference (95% CI)		Within-Groups Effect size—Hedges' g (95% CI)		Between Groups Mean Difference (95% CI)	Between Groups Effect size—Hedges' g (95% CI)
			Yoga	Control	Yoga	Control	Yoga	Control	Yoga	Control	Yoga	Control
Chandwani et al., 2010 [10]	BFI						2.30	2.30	−0.40	0.20	−0.12	0.05	−0.60	−0.17
	(Fatigue)	.94	0.39	0.54	0.80	1.10	(1.60)	(2.20)	[−1.58, 0.78]^a^	[−1.17, 1.57]	[−0.49, 0.24]	[−0.29, 0.39]	[−2.44, 1.24]^c^	[−0.68, 0.34]
	PSQI						7.30	7.10	−0.70	−0.40	−0.15	−0.08	−0.30	−0.06
	(Sleep)	.84	1.52	1.56	1.90	1.95	(3.80)	(3.90)	[−2.46, 1.06]	[−2.15, 1.35]	[−0.51, 0.22]	[−0.42, 0.27]	[−2.79, 2.19]	[−0.57, 0.45]

Cohen et al., 2004 [11]	BFI						3.10	2.80	0.00	0.30	0.00	0.15	−0.30	−0.14
	(Fatigue)	.96	0.48	0.44	1.20	1.10	(2.40)	(2.20)	[−1.03, 1.03]	[−0.72, 1.32]	[−0.47, 0.47]	[−0.35, 0.64]	[−1.76, 1.16]	[−0.84, 0.56]
	PSQI						6.50	7.20	−0.70	0.90	−0.16	0.21	−1.60	−0.37
	(Sleep)	.84	2.00	1.88	2.50	2.35	(5.00)	(4.70)	[−2.82, 1.42]	[−1.23, 3.03]	[−0.62, 0.32]	[−0.29, 0.71]	[−4.62, 1.42]	[−1.07, 0.34]

Danhauer et al., 2009 [13]	FACT- F						30.10	32.70	9.70	−0.10	0.72	−0.01	9.80	0.71
	(Fatigue)	.95	3.00	2.64	6.70	5.90	(13.40)	(11.80)	[2.87, 16.53]^ab^	[−7.45, 7.25]	[0.14, 1.30]*	[−0.50, 0.49]	[−0.27, 19.87]^cd^	[−0.04, 1.47]
	PSQI						8.30	8.60	−2.20	−1.60	−0.46	−0.31	−0.60	−0.12
	(Sleep)	.84	1.88	2.12	2.35	2.65	(4.70)	(5.30)	[−4.65, 0.25]^a^	[−4.14, 0.94]	[−1.00, 0.08]	[−0.81, 0.20]	[−4.14, 2.94]	[−0.86, 0.61]

Littman et al., 2011 [14]	FACT-F						43.10	43.20	1.9	−0.10	0.33	−0.01	2.00	0.25
	(Fatigue)	.95	1.30	1.90	2.90	4.25	(5.8)	(8.50)	[−0.20, 4.00]^a^	[−3.69, 3.49]	[−0.05, 0.71]	[−0.38, 0.36]	[−2.16, 6.16]^c^	[−0.27, 0.78]

Moadel et al., 2007 [15]	FACT-F						34.27	35.88	2.29	−1.11	0.19	−0.08	3.40	0.27
	(Fatigue)	.95	2.75	3.22	6.16	7.21	(12.31)	(14.42)	[−1.11, 5.69]	[−6.50, 4.30]	[−0.10, 0.48]	[−0.45, 0.30]	[−2.66, 9.46]^c^	[−0.21, 0.75]

**Table 2d:** (d)

Study	Outcome measure	Internal consistency (Cronbach *α*)	SEM (Baseline SD × *√*1 − Cronbach's *α*)	Baseline 0.5 SD	Baseline mean (SD)	Within-Groups mean difference (95% CI)	Within-Groups Effect Size—Hedges' g (95% CI)
Bower et al., 2011 [18]	SF36 Gen Health				50.50	14.50	0.61
	(Overall)	.81	9.63	11.05	(22.10)	[1.44, 27.56]^ab^	[0.00, 1.21]*

Danhauer et al., 2008 [19]	FACT-G				75.90	3.50	0.32
	(Overall)	.89	3.55	5.35	(10.70)	[0.33, 6.67]	[0.02, 0.63]*
	FACT-SWB				24.00	0.80	0.18
	(Social)	.69	2.39	2.15	(4.30)	[−0.53, 2.13]	[−0.12, 0.47]
	FACT-FWB				18.00	0.80	0.19
	(Functional)	.80	1.92	2.15	(4.30)	[−0.44, 2.04]	[−0.11, 0.49]
	FACT-EWB				14.00	−0.20	−0.10
	(Emotional)	.74	1.07	1.05	(2.10)	[−0.81, 0.41]	[−0.39, 0.20]
	FACT-PWB				20.00	2.10	0.38
	(Physical)	.82	2.42	2.85	(5.70)	[0.47, 3.73]	[0.07, 0.68]*
	SF12 PCS				41.20	2.30	0.21
	(Physical)	.86	4.27	5.70	(11.40)	[−0.98, 5.58]	[−0.09, 0.50]
	SF12 MCS				48.40	3.60	0.41
	(Mental)	.81	3.75	4.30	(8.60)	[1.04, 6.16]	[0.11, 0.72]**

Duncan et al., 2008 [20]	FACT-G				64.6	10.63	0.69
	(Overall)	.89	4.70	7.30	(14.16)	[4.44, 16.82]^ab^	[0.24, 1.14]**

Galantino et al., 2011 [21]	FACT-B				89.33	16.72	0.81
	(Overall)	.90	6.38	10.09	(20.18)	[−5.07, 28.37]^ab^	[0.14, 1.48]*

Speed-Andrews et al., 2010 [22]	FACT-B				108.20	8.90	0.39
	(Overall)	.90	6.67	10.55	(21.10)	[−1.51, 19.31]^a^	[−0.08, 0.86]
	FACT-SWB				20.70	0.70	0.13
	(Social)	.69	2.95	2.65	(5.30)	[−1.84, 3.24]	[−0.33, 0.58]
	FACT-FWB				19.90	1.80	0.39
	(Functional)	.86	1.83	2.45	(4.90)	[−0.29, 3.89]	[−0.08, 0.86]
	FACT-EWB				15.80	2.10	0.39
	(Emotional)	.69	2.67	2.40	(4.80)	[−0.37, 4.57]	[−0.09, 0.86]
	FACT-PWB				20.90	2.00	0.38
	(Physical)	.81	2.31	2.65	(5.30)	[−0.37, 4.37]	[−0.09, 0.85]
	SF-36 PCS				45.50	3.70	0.32
	(Physical)	.90	3.38	5.35	(10.70)	[−1.41, 8.81]^a^	[−0.14, 0.79]
	SF-36 MCS				44.40	4.20	0.45
	(Mental)	.90	2.85	4.50	(9.00)	[−0.01, 8.41]^a^	[−0.03, 0.93]

Ülger and Yağlı, 2010 [23]	NHP-Total				103.23	−17.32	−0.77
	(Overall)	.70	12.64	11.54	(23.08)	[−26.70, −7.94]^ab^	[−1.26, −0.29]**
	NHP-Social				43.01	−20.56	−0.74
	(Social)	.56	20.01	15.08	(30.16)	[−32.24, −8.88]^ab^	[−1.22, −0.26]**
	NHP-Emotion				61.37	−26.61	−1.15
	(Emotional)	.83	9.68	11.74	(23.47)	[−36.35, −16.87]^ab^	[−1.70, −0.60]***
	NHP-Physical				44.51	−20.41	−0.92
	(Physical)	.72	11.86	11.21	(22.41)	[−29.77, −11.05]^ab^	[−1.43, −0.41]***

**Table 2e:** (e)

Study	Outcome measure	Internal consistency (Cronbach *α*)	SEM (T1 SD × *√*1 − Cronbach's *α*)	Baseline 0.5 SD	Baseline mean (SD)	Within-Groups mean Difference (95% CI)	Within-Groups Effect Size—Hedges' g (95% CI)
Bower et al., 2011 [18]	BDI				15.40	−7.90	−1.00
	(Depression)	.81	3.49	4.00	(8.00)	[−12.20, −3.60]^ab^	[−1.69, −0.32]**

Danhauer et al., 2008 [19]	CES-D 20				12.30	−3.10	−0.36
	(Depression)	.93	2.27	4.30	(8.60)	[−5.66, −0.54]^a^	[−0.66, −0.05]*
	STAI-State				34.20	−2.40	−0.22
	(Anxiety)	.95	2.39	5.35	(10.70)	[−5.57, 0.77]^a^	[−0.52, 0.08]
	PANAS-P				34.70	1.50	0.17
	(Positive Affect)	.87	3.10	4.30	(8.60)	[−1.06, 4.06]	[−0.12, 0.47]
	PANAS-N				15.80	−1.70	−0.35
	(Negative Affect)	.87	1.80	2.50	(5.00)	[−3.14, −0.26]	[−0.65, −0.04]*
	FACIT-Sp				38.70	1.40	0.19
	(Spiritual Well-Being)	.87	2.56	3.55	(7.10)	[−0.74, 3.54]	[−0.10, 0.49]

Duncan et al., 2008 [20]	FACIT-Sp				30.83	5.88	0.60
	(Spiritual Well-Being)	.87	3.65	5.07	(10.13)	[1.93, 9.83]^ab^	[0.16, 1.04]**

Speed-Andrews et al., 2010 [22]	CES-D 10				31.00	−0.20	−0.34
	(Depression)	.85	0.23	0.30	(0.60)	[−0.47, 0.07]	[−0.81, 0.13]
	STAI-State				45.20	1.80	0.25
	(Anxiety)	.85	2.59	3.35	(6.70)	[−1.48, 5.08]	[−0.21, 0.71]

Ülger and Yağlı, 2010 [23]	STAI-State				55.05	−11.70	−1.51
	(Anxiety)	.95	1.75	3.92	(7.83)	[−14.96, −8.44]^ab^	[−2.14, −0.88]***

**Table 2f:** (f)

Study	Outcome measure	Internal consistency (Cronbach *α*)	SEM (Baseline SD × *√*1 − Cronbach's *α*)	Baseline 0.5 SD	Baseline mean (SD)	Within-Groups mean difference (95% CI)	Within-Groups Effect Size—Hedges' g (95% CI)
Bower et al., 2011 [18]	FSI Average				6.3	−3.6	−2.34
	(Fatigue)	.97	0.19	0.55	(1.1)	[−4.44, −2.76]^ab^	[−3.47, −1.22]***
	PSQI				7.1	−0.7	−0.17
	(Sleep)	.84	1.64	2.05	(4.1)	[−2.99, 1.59]	[−0.72, 0.38]

Danhauer et al., 2008 [19]	FACT-F				34.6	2.7	0.22
	(Fatigue)	.95	2.71	6.05	(12.1)	[−0.83, 6.27]^a^	[−0.08, 0.52]

Ülger and Yağlı, 2010 [23]	NHP-S				43.50	−23.47	−0.88
	(Sleep)	.72	15.64	14.78	(29.55)	[−34.69, −12.25]^ab^	[−1.38, −0.38]***

*P < .05, **P < .01, ***P < .001 (Reported between group *P* values).

^a^Within-Group mean difference exceeds 1 SEM.

^b^Within-Group mean difference exceeds Baseline 0.5 SD.

^c^Between-Group mean exceeds control group 1 SEM.

^d^Between-Group mean exceeds control group Baseline 0.5 SD.

Abbreviations—*α*: alpha; SD: standard deviation; CI: confidence interval; SF-36 and SF-12: Medical Outcomes Study Short-Form Health Survey; EORTC QLQ C30: European Organization for Research and Treatment of Cancer-Quality of Life C30; FACT-B: Functional Assessment of Cancer Therapy-Breast; FACT-G: Functional Assessment of Cancer Therapy-General; NHP: Nottingham Health Profile; BDI: Beck Depression Inventory; CES-D 20: Center for Epidemiologic Studies Depression Scale-20 Item; STAI: State/Trait Anxiety Inventory; POMS: Profile of Mood States; PANAS: Positive and Negative Affect Schedule; FACIT-Sp: Functional Assessment of Chronic Illness Therapy-Spiritual Well-Being; HADS: Hospital Anxiety and Depression Scale; BFI: Brief Fatigue Inventory; FSI: Fatigue Symptom Inventory; PSQI: Pittsburgh Sleep Quality Index; FACT-F: Functional Assessment of Cancer Therapy-Fatigue.
